# Prognostic role of aetiological agent vs. clinical pattern in candidates to lead extraction for cardiac implantable electronic device infections

**DOI:** 10.1038/s41598-024-73147-8

**Published:** 2024-12-30

**Authors:** Giulia Massaro, Renato Pascale, Mauro Biffi, Cristian Martignani, Matteo Ziacchi, Andrea Simeone, Raimondo Pittorru, Manuel De Lazzari, Federico Migliore, Igor Diemberger

**Affiliations:** 1 Department of Medical and Surgical Sciences, Institute of Cardiology, University of Bologna, Policlinico S.Orsola-Malpighi, via Massarenti 9, Bologna, 40138 Italy; 2 Infectious Diseases Unit, IRCCS Azienda Ospedaliero-Universitaria di Bologna, Bologna, Italy; 3 Department of Medical and Surgical Sciences, University of Bologna, Bologna, Italy; 4 Dipartimento Cardio-toraco-vascolare, UOC di Cardiologia, IRCCS Azienda Ospedaliero-Universitaria di Bologna, via Massarenti 9, Bologna, 40138 Italy; 5 Department of Cardiac, Thoracic, Vascular Sciences and Public Health, University of Padua, Padua, Italy

**Keywords:** Cardiac implantable electronic device infections, Transvenous led extraction, Aetiological agent, Clinical infection pattern, Cardiac device therapy, Interventional cardiology

## Abstract

Cardiac implantable electronic devices infections (CIEDI) are associated with poor survival despite the improvement in transvenous lead extraction (TLE). Aetiology and systemic involvement are driving factors of clinical outcomes. The aim of this study was to explore their contribute on overall mortality. A prospective study was performed between 2011 and 2021, including all TLE candidates at our regional referral University hospital for CIEDI with microbiological confirmed aetiology. Considering significant predictors of mortality at multivariate Cox regression analyses, a 5-point BOP_2_D score was developed, and it was validated with a prospective cohort from the Padua University. 157 patients were enrolled (mean age 71.3 ± 12.3 years, 81.5% male). *S. aureus* was isolated in 32.5% of patients, and it was more associated with valvular heart disease, systemic infection, and chronic kidney disease. CIEDI pattern was associated with 1-year mortality, with a significantly worse outcome in patients with “cold closed pocket” (CCP). The developed BOP_2_D score presented a 0.807 AUC (95%CI 0.703–0.910, *p* < 0.001) and a good predictive value (OR 2.355, 95%CI 1.754–3.162; *p* < 0.001), and was associated with a progressive increase in mortality with a score > 2. The score validation with the registry from the Padua University (135 patients) retrieved a C-statistic of 0.746 (95%CI 0.613–0.879; *p* = 0.002). Both CCP and *S. aureus* were confirmed as risk factors for mortality in CIEDI patients. This study supports the hypothesis that the infectious process may occur through different mechanisms associated with different infection patterns, and high-risk patients should be considered for specific and aggressive approaches.

## Introduction

The number of patients receiving a cardiac implantable electronic device (CIED) dramatically increased during the last 50 years^1^. Nowadays, CIED infections (CIEDI) represent the most feared complication despite the ongoing improvement in prevention and treatment^2–5^, with a long-term mortality despite transvenous lead extraction (TLE) procedure reaching up to 24%^6,7^. Similarly to infective endocarditis (IE), the main aetiological agents of CIEDI are Gram-positive bacteria with variable incidence among different cohorts. Coagulase-negative *Staphylococci* (CoNS) and *Staphylococcus aureus* (*S. aureus*) are responsible of CIEDI in a large majority of patients (33–69%). Other Gram-positive bacteria as *Enterococcus* spp (0–5%), *Corynebacterium* spp (4–5%), and *Streptococcus* spp (2%) are less represented. Gram-negative bacilli are responsible for 6–7% of CIEDI^8–13^. Available literature on IE in general and on CIEDI specifically supports a worse prognosis of patients with infection due to *S. aureus*, which tends to cause infections with a more severe and acute course.

Another factor reported to be associated with long-term survival after TLE is the pattern of CIEDI involvement^14,15^. Three different patterns were identified: isolated pocket infection, systemic infection with CIED pocket involvement and the so called “cold closed pocket” (CCP), which means infection of transvenous hardware without any clinical or instrumental pocket involvement^14^. In particular, the last pattern was associated with worse long-term outcomes. We aimed our analysis at identifying the relative impact on mortality of bacterial aetiology and clinical pattern in a prospective cohort of patients affected by CIEDI undergoing TLE.

## Methods

### Population data

All patients referred for TLE to Cardiology Unit of IRCCS Azienda Ospedaliero-Universitaria di Bologna and registered in our observational prospective registry were considered for enrolment. The study was conducted according to declaration of Helsinki and Good Clinical Practice guidelines, and the registry was approved by the Ethics Committee of our center (CE-AVEC)^7^.

All patients underwent a specific clinical evaluation carried by experts in CIEDI, including transthoracic (TTE) and transoesophageal echocardiography (TEE), PET/CT scan with fluorodeoxyglucose (F-PET), and microbiological samples [2 sets of blood cultures (BC) in all patients and deep pocket swab in case of pocket dehiscence], preferably before starting antibiotic therapy or after appropriate wash-out. Based on these tests, CIEDI were defined as:

- Pocket infection: infection limited to CIED pocket with local signs of inflammation including erythema, warmth, fluctuance, wound dehiscence, erosion, tenderness, or purulent drainage.
- Systemic infection with CIED pocket involvement: pocket infection extending to the leads, cardiac valve leaflets and/or endocardial surface.
- CCP: infection of transvenous hardware without any clinical or instrumental pocket involvement. Infections with CCP are distinguished from those with clinical signs and/or F-PET uptake suggestive of pocket involvement.

The key aspect to successful treatment of systemic or pocket CIEDI is the complete removal of all parts of the system and transvenous hardware, including abandoned lead fragments^16^. TLE procedures were performed by expert electrophysiologists (according to EHRA consensus)^16^ in a hybrid operating room with active cardiac surgical backup. The specific approach to TLE and CIED reimplantation in our center was described previously^17–19^. All extracted materials underwent culturing after TLE procedure. Empirical antibiotic therapy was started immediately after TLE, when possible. When intra-operative cultures were available, antibiotic therapy was confirmed or modified by a dedicated Infectious Disease (ID) Consultant team. Duration of antibiotic therapy was established according by last guidelines available at time of procedure^16^. Infectious status was monitored through clinical evaluations, inflammatory laboratory tests and BC, when necessary. After discharge, patients were followed up with ambulatory visits at 6 and 12 months.

All patients enrolled in the TLE registry between March 2011 and September 2021 were included in the analyses, with the exclusion of the following conditions:

1. TLE for non-infectious indications (e.g., lead malfunction).
2. Lack of microbiological confirmation of CIEDI diagnosis.
3. Fungal CIEDI.
4. Absence of F-PET scan.

### Microbiology

All patients with CIEDI were evaluated by a dedicated ID Consultant team. To assess systemic infectious involvement before TLE procedure, patients underwent at least 2 BC. Specifically, BC results will be considered related to CIEDI in case of concordance with microbiological samples derived from TLE procedure or if microorganisms were suggestive for CIEDI. CIEDI was defined polymicrobial in case of multiple bacterial growth from intraoperative samples. In case of localized pocket involvement with generator and/or electrode extrusion, deep wound swabs were also collected to establish antibiotic therapy prior the availability of intraoperative samples. Regardless of clinical presentation, all CIED components extracted during TLE procedure were subjected to culture, including generator and distal lead portions. All samples were analysed following the routine diagnostic workflow of our Microbiology laboratory. The routinary bacterial identification was performed using a MALDI-TOF real-time identification system (Bruker Daltonik, Bremen, Germany). The antimicrobial susceptibility testing of bacterial isolates was performed by MicroScan Walkaway-96 automated system (Beckman Coulter, Brea, California, US). Interpretation of Minimum Inhibitory Concentration (MIC) results was performed accordingly to the European Committee for Antimicrobial Susceptibility Testing (EUCAST) or Clinical and Laboratory Standards Institute (CLSI) breakpoints. Patients were considered non-infected if positive result was obtained only from a single sample without any correlation with clinical signs or imaging findings suggesting CIEDI.

### Statistical analysis

Normally distributed continuous variables were expressed as mean and standard deviation, while for non-normally distributed continuous variables, the median and interquartile range were calculated. For these variables, the significance of differences between groups was calculated using Student’s t-test and, when appropriate, the non-parametric Kruskal-Wallis test. Discrete variables were expressed as frequencies and percentages, and significance was calculated using the chi-square test. Survival after TLE was evaluated using the Kaplan-Meier method, and the log-rank test was used to compare survival between different subgroups. The effect of different variables on survival was investigated using Cox regression. ROC curves for the final scoring system was created to asses C statistic among the derivation cohort from the University of Bologna and the testing cohort from the University of Padova. The analysis was carried out using SPSS 23.0 (SPSS Statistics/IBM Corp, Chicago IL, USA), considering a p-value < 0.05 as statistically significant.

## Results

### Population data

266 patients underwent TLE at our third level regional referral center between March 2011 and September 2021. According to previously described criteria, 63 cases were excluded because of non-infectious indications to TLE or not availability of F-PET, 44 due to negative cultures, and 2 because of fungal aetiology. 157 patients were included in the analyses, mean age 71.3 ± 12.3 years, 128 (81.5%) were male (see Table 1). 26 patients (16.6%) were pacemaker-dependent, so after TLE an externalized temporary pacemaker was inserted from the same upper access used for the TLE. Permanent device reimplantation was postponed for 2–4 weeks, if microbiological cultures were confirmed negative in accordance with European guidelines^16^.

**Table 1:** Population data.

Age (years)	71.3 ± 12.3
Male	128 (81.5)
BMI (kg/m^2^)	25.8 ± 4.7
HBP	107 (68.2)
Diabetes	54 (34.4)
RF	52 (33.1)
HD	9 (5.7)
CAD	62 (39.5)
Mitral regurgitation	34 (21.7)
Aortic regurgitation	13 (8.3)
Tricuspid regurgitation	14 (8.9)
Aortic stenosis	12 (7.6)
Mitral stenosis	3 (1.9)
CHD	6 (3.8)
DCM	70 44.6)
HCM	11 (7.0)
Previous sternotomy	25 (15.9)
LVEF	46.3 ± 15.0
NYHA > 2	23 (14.6)
COPD	14 (8.9)
Single-chamber PM	20 (12.7)
Dual-chamber PM	51 (32.5)
CRTP	4 (2.5)
Single-chamber ICD	26 (16.6)
Dual-chamber ICD	19 (12.1)
CRTD	37 (23.6)
AVB	50 (31.8)
SSS	21 (13.4)
CSS	4 (2.5)
Primary prevention	53 (33.8)
Secondary prevention	29 (18.5)
First implantation	56 (35.7)
Replacement	60 (38.2)
Lead revision/upgrade	21 (13.4)
Pocket revision	20 (12.7)
Previous Infection	52 (33.1)
Previous TLE	10 (6.4)
WBC (10^9^/L)	7.2 ± 3.2
Creatinine (mg/dl)	1.51 ± 1.36
CRP (mg/dl)	0.9 [0.3–3.4]
Time to infection (months)	10.1 [2.6–43.1]
Early reimplantation	93 (59.2)
Transvenous	49 (31.2)
Leadless	4 (2.5)
Epicardial	28 (17.8)
Subcutaneous	12 (7.6)
Antibiotic treatment (%)	
Aminoglycoside	8 (5.1)
Amoxicillin	17 (10.8)
Ampicillin	5 (3.2)
Carbapenem	6 (3.8)
Cephalosporin	29 (18.5)
Clindamycin	7 (4.5)
Daptomycin	34 (21.7)
Fluoroquinolone	17 (10.8)
Glycopeptide	15 (9.6)
Oxacillin	22 (14.0)
Piperacillin/tazobactam	7 (4.5)
Rifampicin	26 (16.6)
Trimethoprim/sulfamethoxazole	6 (3.8)

*AVB* atrioventricular block, *BMI* body max index, *CAD* coronary artery disease, *CHD* congenital heart disease, *CRP* c-reactive protein, *CRTD* cardiac resynchronization therapy defibrillator, *CRTP* cardiac resynchronization therapy pacemaker, *CSS* carotid sinus syndrome, *DCM* dilated cardiomyopathy, *HCM* hypertrophic cardiomyopathy, *HD* haemodialysis, *ICD* implantable cardioverter defibrillator, *LVEF* left ventricular ejection fraction, *PM* pacemaker, *pts* patients, *RF* renal failure, *SSS* sick sinus syndrome, *TLE* transvenous lead extraction, *WBC* white blood cells.

**Fig. 1:**
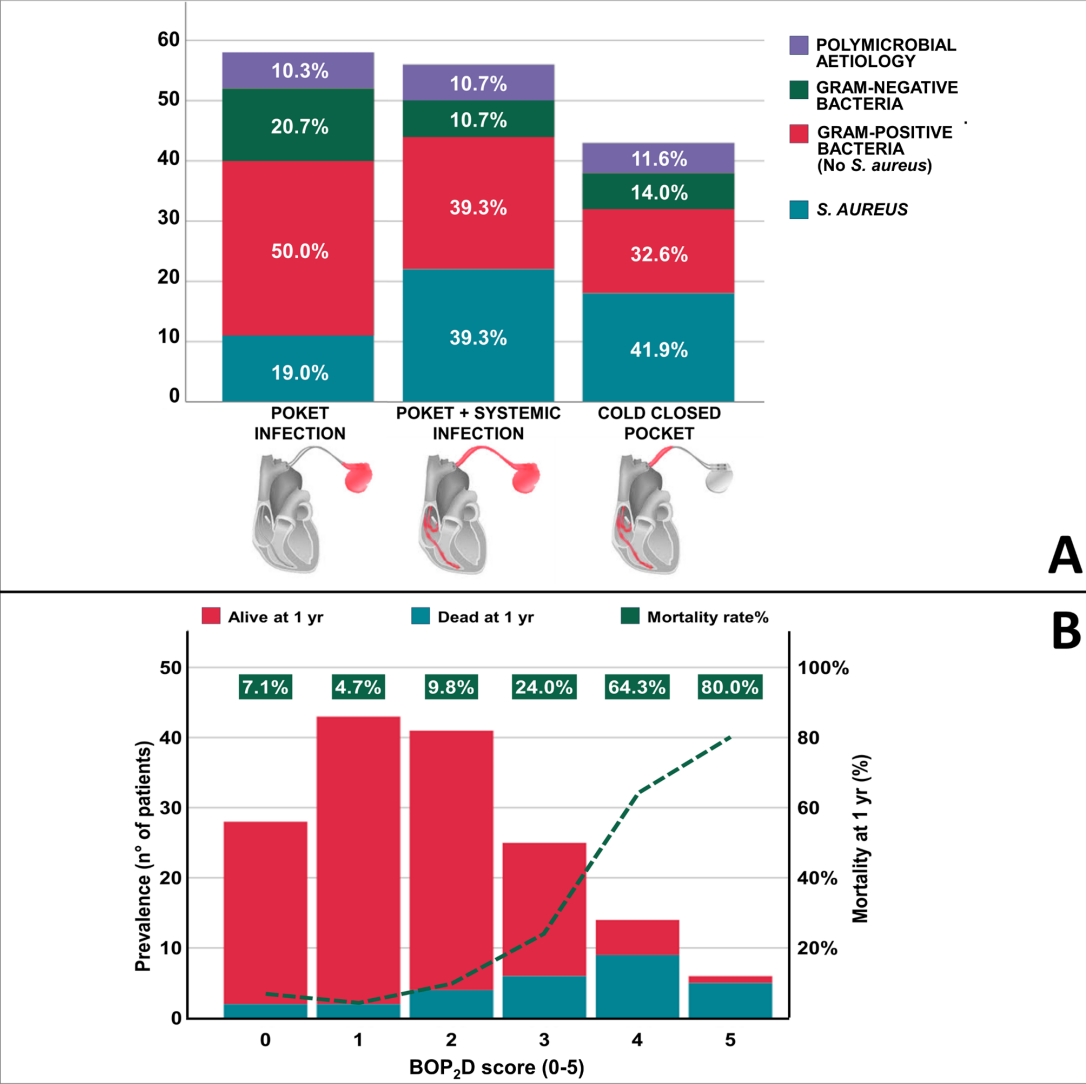
(**A**) Distribution of patients according to CIEDI aetiology and pattern. (**B**) Mortality considering BOP2D score. *n* number, *yr* year.

Considering the pathogen, 51 cases (32.5%) were due to *S. aureus*, 65 (41.4%) to other Gram-positive bacteria, 24 (15.3%) to Gram-negative strains, and 17 patients (10.8%) had polymicrobial infection. Methicillin resistance was observed in 14 (27.5%) of S. aureus strains. Among other Gram-positive bacteria, *S. epidermidis* was most found (37 patients, 56.9%); while considering Gram-negative bacteria, *P. aeruginosa* was the most represented (9 patients, 37.5%). Infections localized to CIED pocket were 58 (36.9%), CIED pocket infections with systemic involvement occurred in 56 patients (35.7%), and CCP was found in 43 cases (27.4%). Figure 1 panel A depicts the distribution of patients according to CIEDI aetiology and pattern. Unsurprisingly, CIEDI due to *S. aureus* were more frequently associated with systemic involvement with/without pocket involvement.

Considering significant predictors of mortality at multivariate Cox regression analyses, we developed the 5-point BOP_2_D score (*S. aureus***B**acteria, Cold Closed P**O**cket, renal im**P**airment, left ventricular **D**ysfunction). The score was validated with an independent prospective registry from the Padua University, collected with similar inclusion criteria of our center, with the exception of F-PET that is less stringently required before TLE, and counting 135 patients (the registry was approved by the local ethical committee and patients provided informed consent before inclusion).

### Microbiological aetiology

A comparison of patients with and without *S. aureus* infection is showed in Table 2. Significant differences were found in the presence of left ventricular dysfunction (*p* = 0.022), valvular heart disease (*p* = 0.008) and infection pattern (*p* = 0.021); in particular, *S. aureus* was associated with the presence of systemic involvement, in contrast with other bacteria that cause more frequently pocket involvement.

**Table 2:** Population data comparing *S. aureus* vs. other microbiological aetiology.

	*S. aureus* 51 (32.5)	Other microbial aetiology 106 (67.5)	*p*
Male	45 (88.2)	83 (78.3)	n.s.
LVEF < 40%	24 (47.1)	31 (29.2)	0.022
CAD	22 (43.1)	40 (37.7)	n.s.
VHD	26 (51.0)	31 (29.2)	0.008
HBP	34 (66.7)	73 (68.9)	n.s.
Diabetes	21 (41.2)	33 (31.1)	n.s.
COPD	6 (11.8)	8 (7.5)	n.s.
ICD	28 (54.9)	54 (50.9)	n.s.
Ghosts	9 (17.6)	25 (23.6)	n.s.
Early reimplantation	25 (49.0)	48 (45.3)	n.s.
Cardiac vegetation	22 (43.1)	33 (31.1)	n.s.
Infection pattern			0.021
Pocket infection	11 (21.6)	47 (44.3)	
Pocket infection with systemic involvement	22 (43.1)	34 (32.1)	
CCP	18 (35.3)	25 (23.6)	

*CAD* coronary artery disease, *CCP* cold close pocket, *COPD* chronic obstructive pulmonary disease, *HBP* high blood pressure, *ICD* implantable cardioverter defibrillator, *LVEF* left ventricular ejection fraction, *VHD* valvular heart disease.

### Role of CCP

Table 3 shows population data according to CIEDI pattern, comparing patients with CCP (43, 27.4%) vs. the remaining population (114, 72.6%). CCP was more frequent in carries of cardiac implantable defibrillators (ICD) (*p* = 0.047). and in patients affected by diabetes (*p* = 0.019). Unsurprisingly patients with CCP had a higher prevalence of cardiac vegetations before TLE (*p* < 0.001) and ghosts^7^ after the procedure (*p* = 0.013).

**Table 3:** Population data comparing CCP vs. other clinical patterns.

	CCP 43 (27.4)	Other patterns 114 (72.6)	*p*
Male	38 (88.4)	90 (78.9)	n.s.
LVEF < 40%	17 (39.5)	38 (33.3)	n.s.
CAD	17 (39.5)	45 (39.5)	n.s.
VHD	19 (44.2)	38 (33.3)	n.s.
HBP	27 (62.8)	80 (70.2)	n.s.
Diabetes	21 (48.8)	33 (28.9)	0.019
COPD	6 (14.0)	8 (7.0)	n.s.
ICD	28 (65.1)	54 (47.4)	0.047
Ghosts	15 (34.9)	19 (16.7)	0.013
Early reimplantation	16 (37.2)	57 (50.0)	n.s.
Cardiac vegetation	34 (79.1)	21 (18.4)	< 0.001
Aetiology			n.s.
*S. aureus*	18 (41.9)	33 (28.9)	
*non-aureus*	20 (46.5)	69 (60.5)	
Polymicrobial	5 (11.6)	12 (10.5)	

*CAD* coronary artery disease, *CCP* cold close pocket, *COPD* chronic obstructive pulmonary disease, *HBP* high blood pressure, *ICD* implantable cardioverter defibrillator, *LVEF* left ventricular ejection fraction, *VHD* valvular heart disease.

### Role of aetiology and CIEDI pattern on overall mortality

**Fig. 2:**
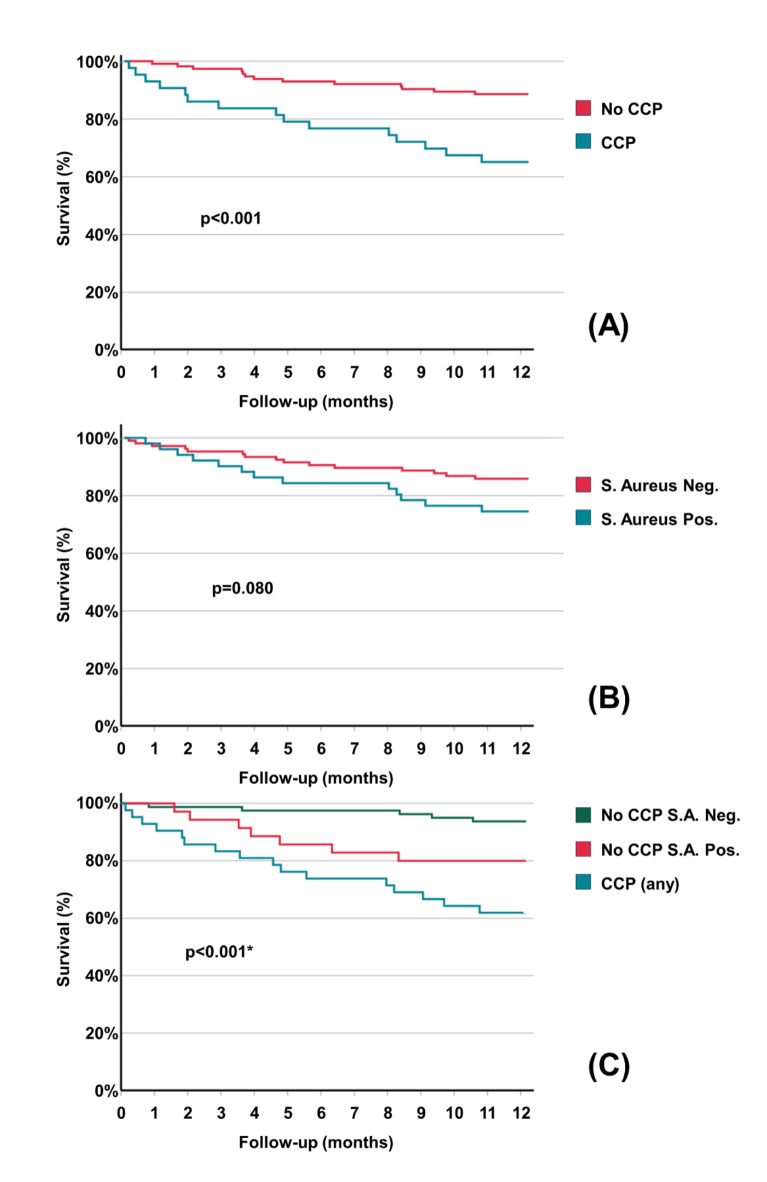
Survival curves considering CCP diagnosis (**A**), microbiological aetiology (**B**), and the association of both variables (**C**). *CCP vs. No CCP & *S. aureus* positive *p* = 0.045; CCP vs. No CCP & *S. aureus* negative *p* < 0.001; No CCP & *S. aureus* positive vs. No CCP & *S. aureus* negative *p* = 0.022 at Log Rank Pairwise. *CCP* cold closed pocket.

At 1-year follow-up, 28 patients (17.8%) died overall. The leading cause of death was heart failure or multi-organ failure (13 patients, 46.4%), followed by any systemic infection (8 patients, 28.6%) and other cardiovascular events (4 patients, 14.3%); in 3 patients (10.7%) it was not possible to define the exact cause of death. CIEDI pattern turned to be significantly associated with 1-year mortality, with a significantly worse outcome in patients with CCP compared to subject affected by pocket infection with/without systemic involvement; *S. aureus* aetiology reached only a tendency (*p* = 0.080) (Fig. 2 panel A and B). Considering that *S. aureus* aetiology was not equally represented in the 3 different CIEDI patterns, we explored the possibility to combine these 2 factors. This combination showed the importance of *S. aureus* aetiology in combination with clinical pattern (Fig. 2 panel C).

Table 4 shows the significant predictors of mortality at Cox regression analyses. When looking at multivariate analyses, the independent predictors of 1-year mortality were the presence of *S. aureus* aetiology and infection patterns (as a combined variable), renal failure (RF, considered as a categorical variable according to our previous publication)^20^ and reduced left ventricular ejection fraction (LVEF). Based on the OR values, we developed a 5-point score calculated as follow: *S. aureus* positivity and CCP pattern 1, moderate RF 1 (i.e., with a calculated creatinine clearance between 60 ml/min and 30 ml/min), severe RF 2 (i.e., with a calculated creatinine clearance < 30 ml/min), and reduced LVEF 1. This score presented a 0.807 AUC (95%CI 0.703–0.910, *p* < 0.001) and a good predictive value (OR 2.355, 95%CI 1.754–3.162; *p* < 0.001), and was associated with a progressive increase in mortality with a score > 2 (Fig. 1 panel B). The BOP_2_D score was subsequently validated with the prospective registry from the Padua University, considering that the only difference in the 2 cohorts was the minor use of F-PET in patients enrolled in Padova. The analysis retrieved a C-statistic of 0.746 (95%CI 0.613–0.879; *p* = 0.002) with the following 1-year mortality according to the calculated score: 0 = 2.6%; 1 = 7.4%; 2 = 17.2%; 3 = 30%, 5 = 50%.

**Table 4:** Predictors of mortality at Cox regression analyses.

	Univariate		Multivariate	
	OR (95%CI)	*P*	OR (95%CI)	*p*
COPD	4.734 (2.082–10.771)	< 0.001		
BMI	0.870 (0.783–0.966)	0.009		
LVEF	0.952 (0.925–0.980)	< 0.001		
LVEF < 40%	3.402 (1.557–7.432)	0.002	2.500 (1.135–5.506)	0.023
Diabetes	2.419 (1.151–5.984)	0.020		
Cardiac vegetations	2.766 (1.308–5.849)	0.008		
WBC	1.133 (1.046–1.227)	0.002		
Neutrophils	1.078 (1.037–1.120)	< 0.001		
RF	2.857 (1.703–4.793)	< 0.001	2.694 (1.601–4.535)	< 0.001
CCP and *S. aureus*	2.642 (1.660–4.206)	< 0.001	2.344 (1.453–3.783)	< 0.001

*BMI* body max index, *CCP* cold closed pocket, *COPD* chronic obstructive pulmonary disease, *LVEF* left ventricular ejection fraction, *RF* renal failure, *WBC* white blood cells.

## Discussion

According to our study, both CIEDI aetiology and clinical pattern have a relevant role as predictors of 1-year mortality. Unsurprisingly, the leading aetiology in our prospective cohort was Gram-positive bacteria^8,21–24^; however, in our study *S. aureus* (32.5%) was more prevalent than CoNS, which are widely recognized in literature as the primary aetiological agent of CIEDI^8^. A recent study, published in 2021, showed that *S. aureus* is progressively emerging as the primary cause of CIEDI^25^. Presence of systemic involvement was found in 62.4% of cases, in contrast with previous data asserting higher preponderance of pocket infections^8,15,24,26^. This result can be related to our strict protocol for the diagnosis of systemic involvement of CIEDI. All patients underwent F-PET before TLE procedure, that provides additional diagnostic value, particularly in the subset of possible CIEDI, and may distinguish between different clinical patterns, as suggested by current guidelines^16^. All extracted materials underwent microbiological culture to isolate aetiological agents, and the exclusion of cases where a specific aetiological agent was not isolated could led to underestimate localized pocket infection if a timely initiation of antibiotic therapy was sufficient to effectively inhibit bacterial growth.

Focusing on *S. aureus*, which is in general associated with high 1-year mortality (20–25%)^27–29^, only few data regarding long-term survival associated with this strain in CIEDI are available. In our cohort, *S. aureus* caused more frequently endovascular involvement compared to other bacteria (*p* = 0.021), which is consistent with the result from European Lead Extraction Controlled (ELECTRa) Registry (42.8% vs. 33.2%; *p* = 0.005)^8,15,24,26^. Moreover, patients with *S. aureus* infection presented a higher prevalence of RF compared to subjects with other microbial infection, and a similar result was reported in a recent study published by Polewczyk et al.^30^. Considering echocardiographic findings it emerged that patients affected by *S. aureus* infection presented more frequently valvular heart disease (51.0% vs. 29.2%; *p* = 0.008) and left ventricular dysfunction (47.1% vs. 29.2%, *p* = 0.022). Considering these results, higher mortality observed in *S. aureus*-infected individuals may be influenced not only by the intrinsic characteristics of the agent, but also by a worse clinical profile of patients with this aetiology^20,31^. In this cohort of patients, infection pattern was the only variable significantly associated with mortality, in particular the diagnosis of CCP (*p* < 0.001). Moreover, *S. aureus* was associated with CCP in 41.9% of cases, while pocket decubitus was more frequently associated with other aetiological agents (81.0%). Considering this result, we could assert that *S. aureus* CIEDI could more frequently be caused by blood dissemination from other infectious sources compared to other bacteria, which generally enter during CIED procedures, hypothesis already proposed by several authors^15,16,32,33^.

Considering significant predictors of mortality, we developed a 5-point score (BOP_2_D score) calculated as follow: *S. aureus* positivity and CCP pattern 1, moderate RF 1, severe RF 2, and reduced LVEF 1. This score presented a 0.807 AUC (95%CI 0.703–0.910, *p* < 0.001) and a good predictive value (OR 2.355, 95%CI 1.754–3.162; *p* < 0.001), and was associated with a progressive increase in mortality with a score > 2. The BOP_2_D score was validated with an independent prospective registry from the Padua University, and the analysis retrieved a C-statistic of 0.746 (95%CI 0.613–0.879; *p* = 0.002).

There are some limitations in this study. The deriving and validation cohorts derived from two different centers, which could have influenced the aetiologic distribution and the management of CIEDI. However, both centers are referral for TLE centralizing patients from a large geographical area, reducing this bias. In addition, the unavailability in both centers of molecular biology diagnostic method, such as 16 S and 18 S ribosomal ribonucleic acid (rRNA) sequencing, might reduce the possibility of microbiological diagnosis; however, these methods are infrequently used in common clinical practice. The rate of negative culture CIEDI is highly variable between centers, commonly arises as a consequence of previous antibiotic administration. To reduce these false negative results, we performed microbiological samples prior to start antibiotic therapy, when possible. In fact, we preferred to exclude from analyses patients with lack of microbiological confirmation of CIEDI because false negative cultures could underestimate not only local infection but even systemic infections due to empirical antibiotic treatment.

## Conclusions

This study confirms the relevant prognostic value of both clinical infection pattern and microbiological aetiology. In view of the constraints of healthcare systems, albeit all patients with CIEDI should undergo TLE as soon as possible, according to our results patients with CCP and/or *S. aureus* aetiology should be prioritized. In view of the impact of CCP on clinical outcomes, initiatives aimed at improving early diagnosis of these patients are warranted. The simple BOP_2_D score could help stratifying the risk of TLE candidates to improve clinical management.

## Data Availability

The datasets used and/or analysed during the current study available from the corresponding author on reasonable request.
